# Blood transcriptomics identifies immune signatures indicative of infectious complications in childhood cancer patients with febrile neutropenia

**DOI:** 10.1002/cti2.1383

**Published:** 2022-05-17

**Authors:** Gabrielle M Haeusler, Alexandra L Garnham, Connie SN Li‐Wai‐Suen, Julia E Clark, Franz E Babl, Zoe Allaway, Monica A Slavin, Francoise Mechinaud, Gordon K Smyth, Bob Phillips, Karin A Thursky, Marc Pellegrini, Marcel Doerflinger

**Affiliations:** ^1^ Department of Infectious Diseases Peter MacCallum Cancer Centre Melbourne VIC Australia; ^2^ 3085 NHMRC National Centre for Infections in Cancer Sir Peter MacCallum Department of Oncology University of Melbourne Melbourne VIC Australia; ^3^ 3085 Sir Peter MacCallum Department of Oncology University of Melbourne Melbourne VIC Australia; ^4^ The Victorian Paediatric Integrated Cancer Service Victoria State Government Melbourne VIC Australia; ^5^ 3085 Infection Diseases Unit Department of General Medicine Royal Children's Hospital Melbourne VIC Australia; ^6^ Walter and Eliza Hall Institute for Medical Research Parkville VIC Australia; ^7^ 3085 Department of Medical Biology The University of Melbourne Melbourne VIC Australia; ^8^ Queensland Children's Hospital Child Health Research Centre The University of Queensland Brisbane QLD Australia; ^9^ 3085 Department of Emergency Medicine Royal Children's Hospital Melbourne VIC Australia; ^10^ Murdoch Children's Research Institute Paediatric Research in Emergency Departments International Collaborative (PREDICT) Melbourne VIC Australia; ^11^ Murdoch Children's Research Institute Melbourne VIC Australia; ^12^ 3085 Department of Paediatrics Faculty of Medicine, Dentistry and Health Sciences University of Melbourne Melbourne VIC Australia; ^13^ Victorian Infectious Diseases Service The Peter Doherty Institute for Infection and Immunity Melbourne VIC Australia; ^14^ Children's Cancer Centre The Royal Children's Hospital Melbourne VIC Australia; ^15^ Unité d'Hématologie Immunologie Pédiatrique Hopital Robert Debré APHP Nord Université de Paris Paris France; ^16^ 3085 School of Mathematics and Statistics University of Melbourne Melbourne VIC Australia; ^17^ Leeds Children's Hospital Leeds General Infirmary Leeds UK; ^18^ Department of Infectious Diseases National Centre for Antimicrobial Stewardship University of Melbourne Melbourne VIC Australia

**Keywords:** bacteraemia, blood transcriptome analysis, febrile neutropenia, immune profiling, paediatric cancer, RNAseq

## Abstract

**Objectives:**

Febrile neutropenia (FN) is a major cause of treatment disruption and unplanned hospitalization in childhood cancer patients. This study investigated the transcriptome of peripheral blood mononuclear cells (PBMCs) in children with cancer and FN to identify potential predictors of serious infection.

**Methods:**

Whole‐genome transcriptional profiling was conducted on PBMCs collected during episodes of FN in children with cancer at presentation to the hospital (Day 1; *n* = 73) and within 8–24 h (Day 2; *n* = 28) after admission. Differentially expressed genes as well as gene pathways that correlated with clinical outcomes were defined for different infectious outcomes.

**Results:**

Global differences in gene expression associated with specific immune responses in children with FN and documented infection, compared to episodes without documented infection, were identified at admission. These differences resolved over the subsequent 8–24 h. Distinct gene signatures specific for bacteraemia were identified both at admission and on Day 2. Differences in gene signatures between episodes with bacteraemia and episodes with bacterial infection, viral infection and clinically defined infection were also observed. Only subtle differences in gene expression profiles between non‐bloodstream bacterial and viral infections were identified.

**Conclusion:**

Blood transcriptome immune profiling analysis during FN episodes may inform monitoring and aid in defining adequate treatment for different infectious aetiologies in children with cancer.

## Graphical abstract

We successfully demonstrate that blood transcriptomic analyses in children with cancer who develop febrile neutropenia may have utility in predicting the cause of fever and in identifying serious infectious complications. This can inform monitoring and aid in defining adequate treatment for different infectious aetiologies in childhood cancer patients.

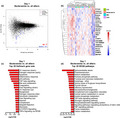

## Introduction

Children with cancer are at increased risk of infection, frequently presenting as fever and neutropenia (FN), due to chemotherapy‐induced immune suppression.^1^ Early (< 24 h) and accurate identification of children at low risk for severe infection during FN is increasingly recognised as important in reducing unnecessary antibiotic exposure and hospital length of stay and improving quality of life.^2^ While some progress has been made in identifying novel blood plasma biomarkers,^3^ next‐generation whole‐genome RNA sequencing technologies investigating the global immune activation landscape as an indicator of infection status during episodes of FN in children with cancer have not been systematically studied. Unique transcriptional signatures in white blood cells indicate pathogenic processes and may be able to distinguish cases with bacterial or viral infection or fever of unknown origin. The blood leukocyte transcriptome during FN therefore reflects aspects of immune status and may have utility in guiding and personalising treatment.^4^, ^5^

Only two studies, one in adults and one in paediatric cancer patients, have been conducted to investigate blood gene expression profiles associated with infections during episodes of FN.^6^, ^7^ Limitations of these studies include small cohort sizes and cross‐sectional and retrospective analyses rather than prospective longitudinal follow‐up and restricted depth of analysis. In paediatric cancer patients, RNAseq analysis of 43 FN episodes identified a panel of two genes to be significantly differentially expressed in bacterial infection compared to controls, but the analysis was not extended to include viral infection or co‐infection.^6^ The paediatric study also concluded that the blood transcriptome was not suitable for determining the aetiology of FN due to the lack of sufficient circulating immune cells impacting the quality of gene expression analysis.^6^

The Australian Predicating Infectious ComplicatioNs In Children with Cancer (PICNICC) study was a large multisite, prospective cohort study designed to validate existing paediatric FN clinical decision rules (CDRs) and to identify novel biomarkers and immune profiles that predict severe infection.^8^, ^9^ In the same cohort of patients, we have shown that procalcitonin (PCT) and interleukin (IL)‐10 may enhance the accuracy of existing CDRs for the prediction of bacterial infection.^10^ In this exploratory study we compared the transcriptional profile of peripheral blood mononuclear cells (PBMCs) from children with cancer and FN with documented infection and unexplained fever. We also investigated how the transcriptomes changed over the 24‐h period post admission and initiation of therapy.

## Results

### Patient characteristics and infection outcomes

Blood PBMC samples were collected on Day 1 from 80 of 553 FN episodes, occurring in 64 patients, enrolled at RCH and QCH as part of the Australian PICNICC study (RCH *n* = 68; QCH *n* = 12). There were 12 patients with two FN episodes, and two patients with three FN episodes. In 31 of these FN episodes, a second sample was collected on Day 2 (between 8 and 24 h from presentation). The median time between Day 1 and Day 2 samples was 21.5 h (IQR: 18.3–23.3 h, range 6.8–30.3). All FN episodes had blood cultures collected prior to the first dose of antibiotics. In seven patients, insufficient PBMC RNA precluded analysis, and therefore transcriptional profiling was conducted across 73 FN episodes (28 had Day 2 samples).

Demographic and outcome data are included in Table 1. Overall, there was no significant difference in baseline demographics, causes of infection and clinical complications (as defined in Ref. 11 and in Supplementary table 1) between the cohort where transcriptional profiling was performed and the total cohort. Of those with diagnosed bacteraemia, three were Gram‐positive and six were Gram‐negative organisms. In the non‐bloodstream microbiologically defined infection (MDI) group, seven were bacterial and 12 were viral infections.

**Table 1:** Demographic and outcome data

	FN cohort without blood sample taken, *n* = 473	FN cohort with PBMC transcriptome on Day 1, *n* = 73	*P*‐value
Median age, years (IQR)	6.0 (3.4–11.3)	4.3 (2.9–9.3)	0.06
Female, *n* (%)	223 (47.1)	34 (46.5)	> 0.99
Diagnosis, *n* (%)			
Haematological malignancy	260 (55.0)	48 (65.8)	0.099
Solid tumour	213 (45.0)	25 (34.2)	
Severe sepsis at presentation, *n* (%)	4 (0.9)	1 (1.4)	0.51
ICU admission, *n* (%)	15 (3.2)	2 (2.7)	> 0.99
Infection diagnosis^11^			
Bacteraemia	66 (14.0)	9^a^ (12.3)	0.087
Non‐bloodstream MDI (bacterial, viral)	90 (19.0)	19 (26.0) (bacterial = 7, viral = 12)	
CDI	44 (9.3)	12^b^ (16.4)	
Unexplained fever	273 (57.7)	33 (45.2)	
Median LOS, days (IQR)	6.0 (3.1–13.3)	5.0 (3.0–7.5)	0.042
Death	2 (0.4)	0	> 0.99

FN, febrile neutropenia; IQR, interquartile range; ICU, intensive care unit; MDI, microbiologically defined infection; CDI, clinically defined infection; unexplained fever, fever of unknown cause; LOS, length of stay.

^a^ Gram‐negative (*Klebsiella pneumoniae*/*Pseudomonas aeruginosa*, *Escherichia fergusonii*, *Neisseria lactamica*, *Pseudomonas* species, *Escherichia coli, Fusobacterium nucleatum*) and Gram‐positive (*Staphylococcus epidermidis*, *Enterococcus faecium, Enterococcus faecalis*).

^b^ Included seven upper respiratory tract infections; two skin and soft tissue infections; two gastroenteritis.

There were no differences in total white blood cell (WBC) count and absolute neutrophil count (ANC) or proportion of cell populations, across episodes with bacteraemia, MDI, clinically defined infection (CDI) and unexplained fever (Supplementary table 2).

### Global transcriptional downregulation of immune signalling pathways distinguishes infectious and non‐infectious causes of fever, and these differences diminish with treatment

**Figure 1:**
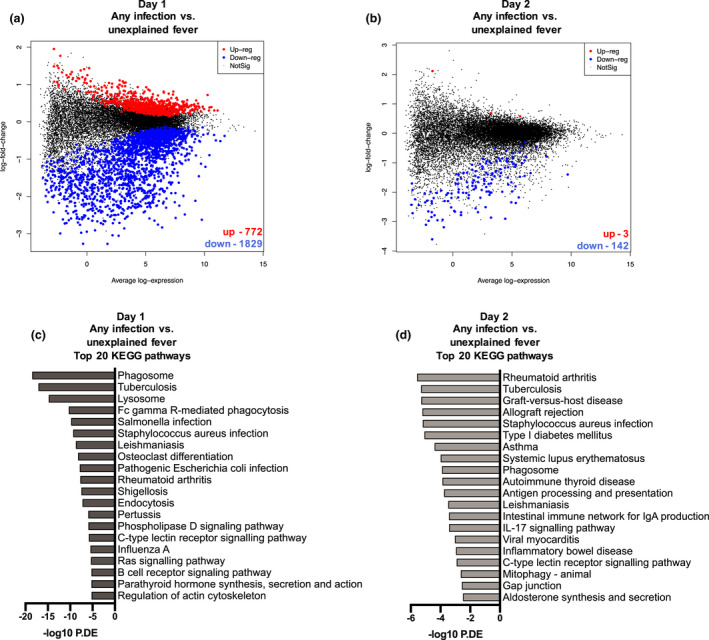
Differential gene expression and pathway analysis comparing infection group to unexplained fever on Day 1 and Day 2. **(a)** Mean–difference (MD) plot showing the 1829 down‐regulated (blue) and 772 up‐regulated (red) DE genes in PBMCs from FN episodes with ‘any infection’ (bacteraemia, MDI and CDI combined) versus unexplained fever at time of hospital admission (Day 1). **(b)** MD plot showing the 142 down‐regulated (blue) and three up‐regulated (red) DE genes in PBMCs from FN episodes with ‘any infection’ (bacteraemia, MDI and CDI combined) versus unexplained fever on Day 2. **(c)** Top 20 KEGG pathways over‐represented when comparing DE genes in PBMCs from FN episodes with ‘any infection’ (bacteraemia, MDI and CDI combined) versus unexplained fever at time of hospital admission (Day 1). (**d)** Top 20 KEGG pathways over‐represented when comparing DE genes in PBMCs from FN episodes with ‘any infection’ (bacteraemia, MDI and CDI combined) versus unexplained fever on Day 2.

After filtering and normalization, 16 559 genes were included in the differential gene expression analyses of any infection versus unexplained fever. On Day 1, 2601 genes were differentially expressed in episodes with any infection (i.e. bacteraemia, other MDI or CDI) compared to episodes with unexplained fever with 772 genes up‐regulated and 1829 genes down‐regulated (Figure 1a). On Day 2, 145 genes were differentially expressed in episodes with any infection compared to unexplained fever, with three genes up‐regulated and 142 genes down‐regulated (Figure 1b).

Kyoto Encyclopaedia of Genes and Genomes (KEGG) pathway analysis and Gene Ontology (GO) were performed to identify signalling pathways associated with the genes differentially expressed between episodes with any infection and episodes with unexplained fever. On Day 1, the top 20 KEGG pathways included those implicated in phagosome and lysosome formation, as well as pathways implicated in response to a diverse spectrum of pathogens including bacteria (*Mycobacterium tuberculosis*, *Salmonella* spp., *Staphylococcus* spp., *Escherichia coli*) and parasites (Leishmania) (Figure 1c). On Day 2, pathways that involved phagocytosis and pathogen response (i.e. *M. tuberculosis*, *Staphylococcus aureus*, Phagosome) as well as an array of autoimmune responses (rheumatoid arthritis, Type 1 diabetes, asthma, systemic lupus and autoimmune thyroid disease) were identified (Figure 1d).

The most common gene ontology terms over‐represented in the differentially expressed genes on Day 1 included vesicle‐mediated immune responses (Supplementary figure 1a) and on Day 2 signal transduction and biological regulation (Supplementary figure 1b).

Genes that were up‐ or down‐regulated on Day 2 were also differentially expressed on Day 1 between episodes with any infection and episodes with unexplained fever (Supplementary figure 1c). A common set of 129 genes were differentially expressed on both Day 1 and Day 2. Of these, two genes were up‐regulated and 127 were down‐regulated (Supplementary figure 1d). Among the top differentially expressed genes identified in all comparisons across both days were ATF3 (activating transcription factor 3), TNFRSF21/DR6 (death receptor 6) and SLC4A3 (solute carrier 4A3 – anion exchange protein 3). Only one gene (INSRR *–* insulin receptor–related receptor) was exclusively differentially expressed in Day 2 samples.

### Gene signature can distinguish bacteraemia versus no bacteraemia FN episodes on admission

**Figure 2:**
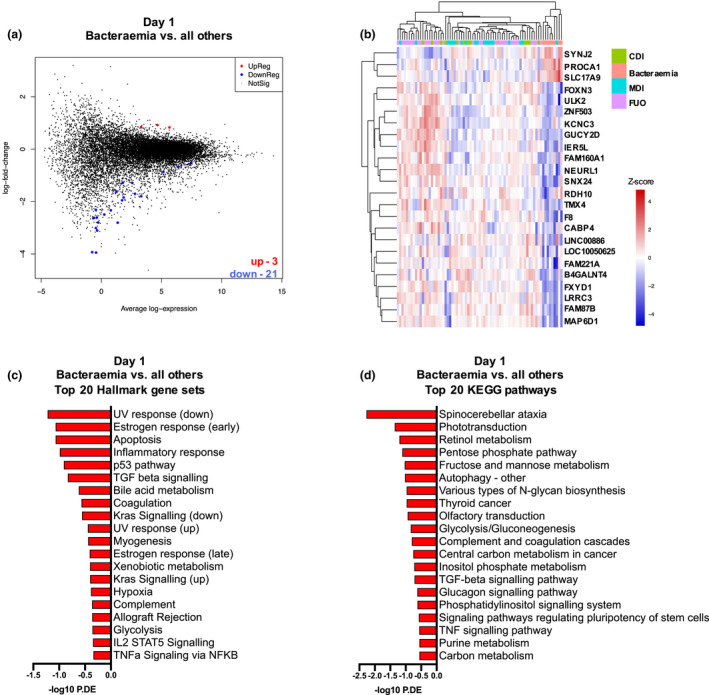
Differential gene expression and pathway analysis of bacteraemia versus all other causes of FN on Day 1. **(a)** MD plot showing the 21 down‐regulated (blue) and 3 up‐regulated (red) DE genes in PBMCs from FN episodes with bacteraemia versus ‘all other’ causes at the time of hospital admission (Day 1). **(b)** Heatmap of the 24 significantly differentially expressed genes (*P *< 0.05) in in PBMCs from FN episodes with bacteraemia versus ‘all other’ causes at the time of hospital admission (Day 1). Colour code indicates *Z*‐score. **(c)** Top 20 Hallmark gene sets over‐represented when comparing DE genes in bacteraemia versus ‘all other’ causes on Day 1. (**d)** Top 20 KEGG pathways over‐represented when comparing DE genes in bacteraemia versus ‘all other’ causes on Day 1.

We next compared the nine FN episodes with bacteraemia to all other FN episodes. After filtering and normalization, 15 518 genes were included in the differential gene expression analyses comparing different types of infection. On Day 1, 24 genes were differentially expressed, of which three were up‐regulated and 21 down‐regulated (Figure 2a and b, Supplementary table 3). A Hallmark gene set analysis, based on the top 50 genes, found that the top 20 pathways were involved in cell death (apoptosis, p53 pathway), inflammatory processes (i.e. signalling via TGF‐beta, IL2‐STAT5, TNF), metabolism (i.e. bile acid and glycolysis) and coagulation and complement activation (Figure 2c). These findings aligned with a KEGG pathway analysis (Figure 2d).

We next explored whether the 24 unique genes identified in episodes with bacteraemia versus all other episodes could also differentiate bacteraemia from other types of infection on Day 1. Comparison of episodes with bacteraemia and episodes with CDI identified nine differentially expressed genes, comparison of bacteraemia and bacterial non‐bloodstream MDI identified four genes and comparison of bacteraemia and viral MDI identified four genes (Supplementary figure 2a–c). Two genes, retinol dehydrogenase (RDH10) and sortin nexin 24 (SNX24), were uniquely differentially expressed and down‐regulated in bacteraemia episodes across all comparisons (Supplementary figure 2d).

### Blood transcriptional changes on admission differs among bacterial infection, viral infection and unknown causes of FN episodes

**Figure 3:**
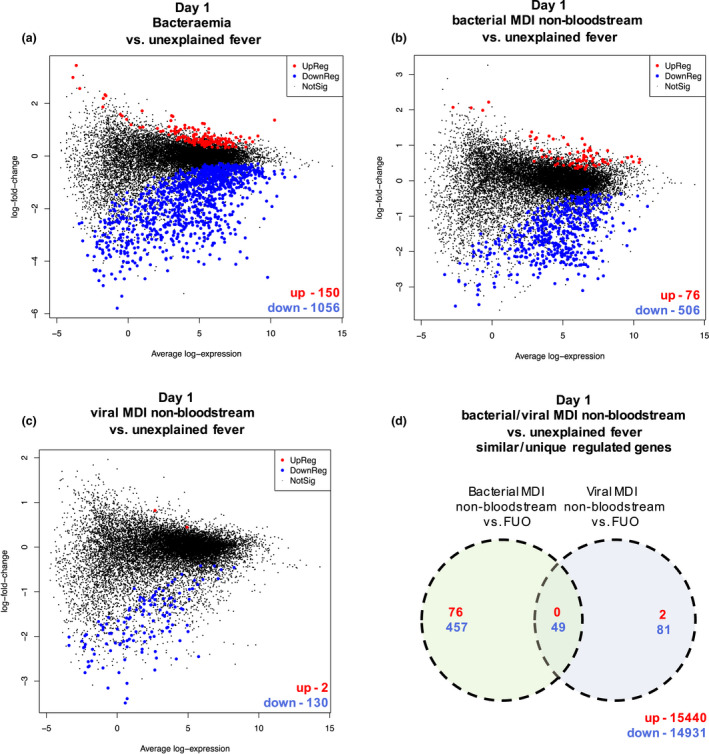
Differential gene expression analysis of comparing different MDI infections versus unexplained fever on Day 1. **(a)** MD plot showing the 1056 down‐regulated (blue) and 150 up‐regulated (red) DE genes in PBMCs from FN episodes with bacteraemia versus unexplained fever at time of hospital admission (Day 1). **(b)** MD plot showing the 506 down‐regulated (blue) and 76 up‐regulated (red) DE genes in PBMCs from FN episodes with non‐bloodstream bacterial MDI versus unexplained fever at time of hospital admission (Day 1). **(c)** MD plot showing the two down‐regulated (blue) and 130 up‐regulated (red) DE genes in PBMCs from FN episodes with viral non‐bloodstream MDI versus unexplained fever on Day 2. **(d)** Common and unique DE genes in non‐bloodstream bacterial MDI versus unexplained fever and viral MDI versus unexplained fever FN episodes at the time of admission (Day 1). Unique DE genes are indicated in the respective circles, while all common DE genes are indicated at the bottom right of the diagram (red, up‐regulated; blue, down‐regulated).

We additionally investigated whether bacteraemia, non‐bloodstream bacterial and viral infections would elicit different transcriptional changes in immune signalling when compared to episodes of unexplained fever. We identified 1206 differentially expressed genes when comparing bacteraemia to unexplained fever episodes (150 up‐regulated, 1056 down‐regulated) (Figure 3a). A comparison of non‐bloodstream MDI to unexplained fever revealed 582 differentially expressed genes (76 up‐regulated, 506 down‐regulated), and 132 genes differentially expressed between episodes of viral MDI and unexplained fever (2 up‐regulated, 130 down‐regulated) (Figure 3b and c). Of these 533 genes were uniquely differentially expressed in bacterial non‐bloodstream MDI and 83 genes were uniquely expressed in viral MDIs, while 49 genes were commonly expressed across episodes with both bacterial and viral MDI compared to episodes with unexplained fever (Figure 3d). No significant differentially expressed genes were found in direct comparison of episodes with bacterial non‐bloodstream infection or viral infection.

### Longitudinal analysis in bacteraemia episodes shows treatment‐associated immune and metabolic restoration

**Figure 4:**
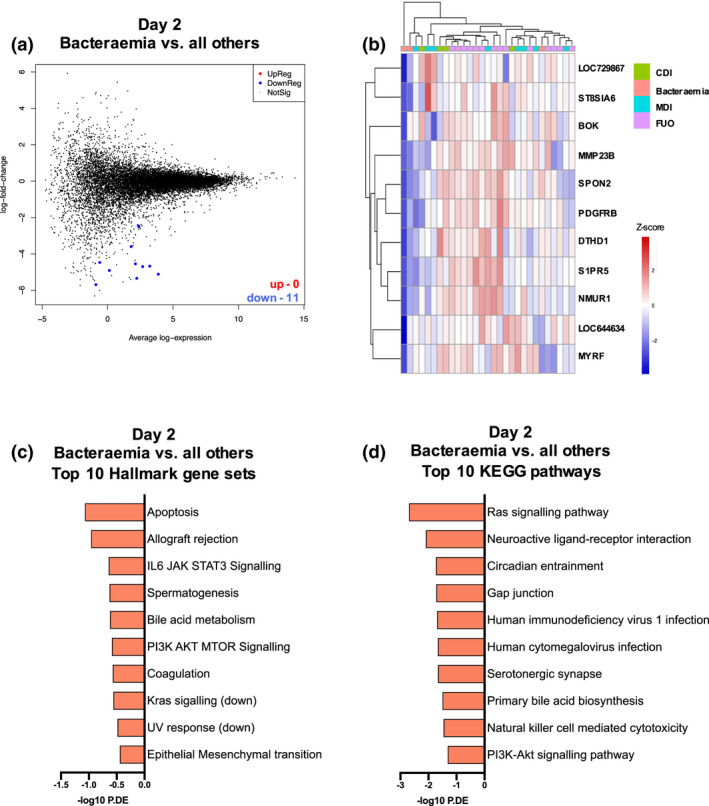
Differential gene expression and pathway analysis of bacteraemia versus all other causes of FN on Day 2. **(a)** MD plot showing the 11 down‐regulated (blue) DE genes in PBMCs from FN episodes with bacteraemia versus ‘all other’ causes on Day 2. **(b)** Heatmap of 11 significantly differentially expressed genes (*P *< 0.05) in PBMCs from FN episodes with bacteraemia versus ‘all other’ causes on Day 2. Colour code indicates the *Z* score. **(c)** Top 10 Hallmark gene sets over‐represented when comparing DE genes in bacteraemia versus ‘all other’ causes on Day 2. **(** **d)** Top 10 KEGG pathways over‐represented when comparing DE genes in bacteraemia versus ‘all other’ causes on Day 2.

On Day 2, 11 genes were differentially expressed between episodes with and without bacteraemia, all of which were down‐regulated (Figure 4a and b, Supplementary table 4). The top 10 Hallmark gene sets and top 10 KEGG pathways, included apoptosis, bile acid metabolism and PI3K‐Akt signalling (Figure 4c and d).

Comparison of episodes with bacteraemia and episodes with either unexplained fever, CDI or MDI on Day 2 identified five genes, including BOK (BCL2 family apoptosis regulator BOK), which were uniquely differentially expressed in bacteraemia episodes across all comparisons (Supplementary figure 3a).

**Figure 5:**
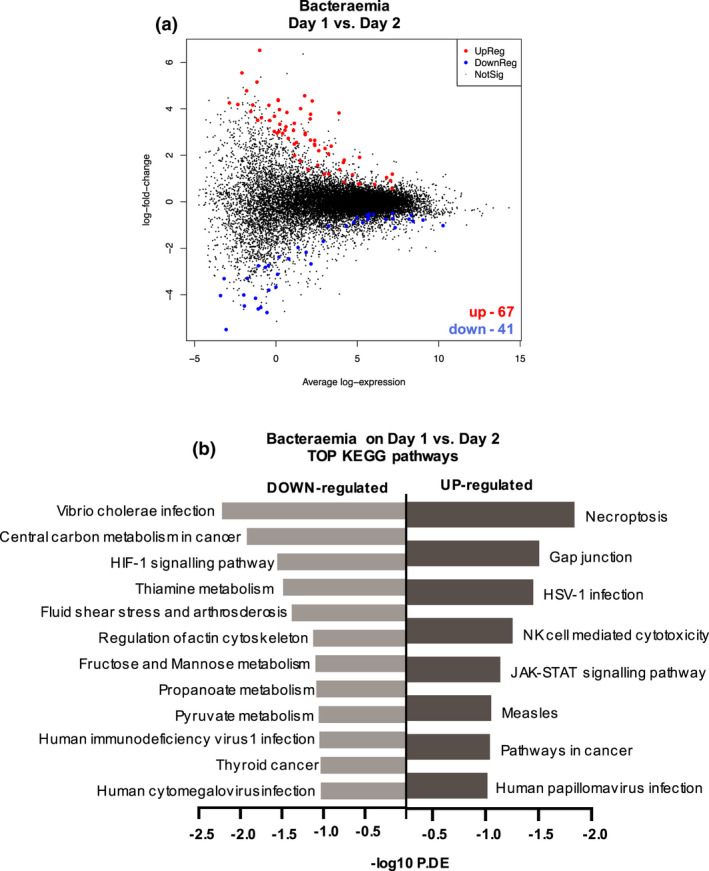
Differential gene expression and hallmark gene set pathway analysis of bacteraemia episodes comparing blood collected on Day 1 to blood collected on Day 2. **(a)** MD plot showing the 67 up‐regulated (red) and 41 down‐regulated (blue) DE genes in PBMCs from FN episodes in bacteraemia episodes on Day 1 and Day 2. **(b)** Top KEGG pathways up‐regulated or down‐regulated when comparing DE genes in bacteraemia episodes on Day 1 and Day 2.

The gene signatures distinguishing bacteraemia from all other causes of FN on Day 1 (24 genes, see Figure 2a and b) and Day 2 (11 genes, see Figure 4a and b) were compared, and no overlapping genes were identified (Supplementary figure 3b). Comparison of differential gene expression comparing bacteraemia on Day 1 versus Day 2 identified 108 genes (67 up‐regulated, 41 down‐regulated) (Figure 5a). The top 20 KEGG pathways included eight that were up‐regulated in Day 1 bacteraemia episodes. Amongst those pathways were necroptosis, NK cell–mediated cytotoxicity and JAK‐STAT–mediated immune signalling (Figure 5b). In contrast, 12 pathways were up‐regulated in Day 2 bacteraemia episodes, including a range of metabolic pathways involving carbon, thiamine propanoate and pyruvate metabolism (Figure 5b).

## Discussion

This exploratory study of the blood transcriptome of PBMCs in children with cancer and FN identified specific profiles that may aid in categorizing causes of fever on presentation and Day 2 of hospital admission. Unique gene profiles differentiating episodes with and without infection and more specifically with and without bacteraemia were identified. These profiles were independent of total WCC and ANC, rather the differences observed between infectious and presumed non‐infectious causes of FN were due to more subtle changes in immune cell signalling not absolute cell numbers.^4^

We identified almost 500 genes that were differentially expressed and distinguished infectious causes of FN from unexplained fever. Amongst the top differentially expressed genes was ATF3 (activating transcription factor 3), a transcription factor that modulates immune response by negatively regulating inflammatory genes, calcium signalling and lysosome formation^12^, ^13^, ^14^ and was shown to provide protection against bacterial infections.^15^ Genes involved in phagocytosis and lysosome/vesicle formation are key pathways involved in early innate responses to infection and were also differentially regulated between infectious and non‐infectious causes of FN in our study. This is in keeping with a study in adult cancer patients with FN which showed that vesicle‐mediated transport and cytokines may help distinguish bacterial causes of fever from other causes.^7^

Our transcriptomic data showed that unexplained fever can be distinguished from other causes of FN based on immune gene activity suggesting that the fundamental cause of these fevers may not be due to undiagnosed or resolving infection. Amongst the different infective causes of FN, transcriptional gene profiling was able to distinguish bacteraemia from other causes including viral infection. This has important clinical implications as the risk of invasive bacterial infection in children with FN drives broad‐spectrum antibiotic exposure. We detected the highest amount of unique differentially expressed genes between bacteraemia and unexplained fever episodes (> 1200 genes). Interestingly, bacterial non‐bloodstream MDI had more differentially expressed genes (582 genes) than viral MDI (132 genes) when compared to unexplained fever, potentially indicating less profound systemic immune responses and immune gene signatures in viral infections. While respiratory viruses such as rhinovirus, influenza virus and respiratory syncytial virus are commonly detected during episodes of FN, it remains unclear whether these viruses are always the primary cause of fever.^16^ Our transcriptional analysis was able to distinguish viral and bacterial causes (i.e. bacteraemia and bacterial non‐bloodstream infections). A direct comparison of viral and bacterial non‐bloodstream infections did not identify differentially expressed genes. However, pathway analysis revealed that, while overall similar pathways were dysregulated when comparing bacterial or viral FN episodes to unexplained fever, the changes were more dramatic in the former comparison and more subtle in the latter comparison.

The present ‘gold standard’ diagnostic test for bacteriaemia is blood culture which can take up to 48 h to identify a causative pathogen.^17^ Given that we identified a unique gene signature in bacteraemic patients compared to those with unexplained fever and unique signatures associated with MDI compared to unexplained fever episodes (Figure 3), we further dissected whether particular patterns of differentially expressed genes at the time of hospital admission could distinguish children who have high‐risk infections. Among the gene signature consisting of 24 genes that were differentially expressed in FN episodes with bacteraemia compared to all episodes without bacteraemia were genes shown to have roles in calcium signalling (*CABP4*) and phospholipid metabolism (*ULK2*). Calcium and phospholipids are required for initiation of coagulation and platelet activation.^18^ This is of particular interest as calcitonin, the active form of procalcitonin (PCT) and a promising blood plasma biomarker for risk stratification in FN, is important in regulating calcium homeostasis in steady state and during severe infection.^19^, ^20^

Of the 24 genes identified in episodes with bacteremia, two genes (SNX24 and RDH10) were uniquely differentially expressed (both down‐regulated) when bacteraemia was compared to each individual cause of FN. Retinol dehydrogenase (RDH10) is a key enzyme in retinoic acid (RA) synthesis.^21^ Retinoic acid was shown to decrease inflammatory processes^22^ and improve immunocompetence in sepsis^23^, ^24^ and is known to regulate bile acid homeostasis which was shown to predict outcome in critically ill patients.^25^ Taken together, this supports the relevance of retinoic acid and its key enzyme retinol dehydrogenase as potential biomarkers to discriminate causes of FN at the time of hospital admission.

Our unique capacity to utilise a prospective longitudinal study design allowed us to compare samples collected at the time of admission to those collected on Day 2 after patients were commenced on empiric FN antibiotics. This analysis revealed that the main signalling pathways that were differentially regulated on Day 2 were involved in aspects of cellular signalling, regulation and communication as well as response to organic substances, Longitudinal analysis of transcriptional profiles in bacteraemia episodes from Day 1 versus Day 2 identified that while genes for apoptosis, bile acid metabolism and coagulation were similarly down‐regulated on Day 2, pathways reported to be responsible for immune recovery and metabolic restoration, such as thiamine metabolism and HIF‐1a signalling^26^, ^27^ were over‐represented on Day 2. Overall, this suggests that treatment may alter the transcriptional profiles over time and the changes observed may indicate treatment response.

BOK was identified as one of the differentially expressed genes that distinguished bacteraemia from all other causes of FN in individual head‐to‐head comparisons on Day 2 and could thus be an interesting biomarker gene. BOK’s role in programmed cell death and apoptosis is still not clearly defined,^28^ but a recent report identified a role for this gene in the control of uridine metabolism.^29^ Interestingly, BOK has been proposed to play an essential role in regulating mitochondrial calcium levels.^30^ We therefore speculate that BOKs overlapping functions in key pathways impacted by bacteraemia – cell death, metabolism, and calcium signalling – might cause its differential expression during FN episodes with underlying bacteraemia.

Although our cohort had only nine bactaeremia episodes, our study is the largest transcriptomic analysis of children with cancer and FN. We obtained sufficient RNA from > 90% of FN episodes and there was no trend towards insufficient RNA in any of the groups, thus overcoming limitations of a previously reported study.^6^ Clinical data informing this study were also collected prospectively as part of a larger cohort study with international definitions of bacteraemia, MDI and CDI used. While microbiological and molecular testing was done at the discretion of the treating clinician and therefore may have missed some infection diagnoses, all patients did have pre‐antibiotic blood cultures taken.

As this was an exploratory study, analyses were performed on stored PBMCs to facilitate coordinated RNA extraction and processing. An independent replication cohort would be useful in validating our data and in defining a minimum set of differentially expressed genes that can discriminate bacteraemia from all other causes of FN. Future prospective studies could then be used to ascertain the positive and negative predictive values of these key transcriptional changes in diagnosing bacteriaemia in FN patients. The clinical utility of gene expression profiling is best exemplified in the diagnosis, characterization, and prognostic evaluation of many cancers.^31^ Further work is needed to correlate whether the immune profiles identified using RNAseq are reflected in the plasma proteome or metabolome. This could aid in identifying novel blood biomarkers that are more readily translatable for diagnostic point‐of‐care test.

## Conclusion

Collectively, our data showed that transcriptomic analyses performed on PBMCs collected from children with cancer who develop FN may have utility in predicting the cause of fever. Blood collected at presentation showed transcriptional signatures that allowed differentiation of bacteraemic causes of fever from other causes of fever. Interestingly, this signature is dampened after the institution of appropriate antibiotics and is replaced with different signatures indicating a degree of immune recovery. We postulate that transcriptomic analysis can potentially identify the cause of fever in neutropenic children with cancer and sequential analyses may provide evidence that the correct treatment has been initiated. It will be important to follow up our findings with further studies in replication cohorts and to understand their accuracy in predicting the cause of FN in children with cancer.

## Methods

### Patient recruitment and blood sample collection

The present blood immune transcriptome study was embedded in the Australian Predicating Infectious ComplicatioNs In Children with Cancer (PICNICC) study (Australian New Zealand Clinical Trials Registry 12616001440415).^8^ Recruitment for the transcriptomic analysis was conducted at two of the eight study sites^8^: Royal Children’s Hospital (RCH), Melbourne, and Queensland Children’s Hospital (QCH), Brisbane. The analysis of plasma abundance of 33 cytokines, CRP and PCT in the same cohort of patients is reported elsewhere.^10^

Children with solid‐organ cancer or leukaemia on active treatment and who presented to the emergency department with FN were included. Fever was defined as a single temperature ≥ 38°C, and neutropenia was defined as an absolute neutrophil count (ANC) < 1000 mm^−3^. Children who had a hematopoietic stem cell transplant (HSCT) in the three months prior to recruitment and those already receiving antibiotics were excluded. Demographic and clinical data including outcomes were prospectively collected from electronic and paper‐based records and entered into REDCap.^32^

Blood from eligible patients was collected at two time points: FN onset (within 0–4 h of ED presentation and prior to 1st dose antibiotic) and Day 2 (within 8–24 h of ED presentation). Blood was collected in EDTA tubes and processed by the cancer centre’s tissue banks at RCH and QCH. Within 2 h of sample collection, plasma was separated from erythrocytes and PBMCs using Ficoll density gradient centrifugation and PBMCs stored in RNAlater at −80°C until thawed for RNA extractions and downstream processing.

The causes of fever were prospectively classified as microbiologically documented infection (MDI), clinically documented infection (CDI) or unexplained fever according to international consensus definitions^11^ (see Supplementary table 1). Bacteraemia, with a known pathogen or a common commensal cultured on two or more occasions, was considered a subset of MDI.^11^ Microbiological and other investigations were performed according to site FN guidelines. Across both sites this included at least one blood culture set (all patients), urine and nasal swabs for PCR or culture where applicable, chest X‐ray, stool culture with *Clostridioides difficile* toxin assay and viral PCR and skin or wound swab for culture and viral PCR where applicable.

Patients were managed according to hospital FN guidelines which included early administration of an anti‐pseudomonal beta‐lactam or cephalosporin after blood cultures were taken.

For the transcriptome analysis FN episodes were grouped into (1) ‘any infection’ versus unexplained fever; (2) bacteraemia, MDI or CDI versus unexplained fever; (3) bacterial non‐bloodstream MDI or viral MDI versus unexplained fever; and (4) bacteraemia versus non‐bacteraemia.

### Transcriptional profiling

The PBMCs were stored in RNAlater™ Stabilization Solution (CAT# AM7020; Thermo Fisher Scientific, Waltham, MA, USA) and RNA extraction was performed using the Isolate II RNA mini kit according to manufacturer’s instruction (Cat# BIO‐52072; Meridian Bioscience, Cincinnati, OH, USA).

An input of 10–100 ng of total RNA was prepared and indexed for illumina sequencing using the TruSeq RNA sample Prep Kit (Cat# RS‐122‐2001; Illumina, San Diego, CA, USA) with RiboGlobin depletion as per manufacturer’s instruction. Each library was quantified using the Agilent Tapestation (using RNA ScreenTape [Cat# 5067‐5576] on a 2200 TapeStation system (Cat# G2964AA; Agilent Technologies, Waldbrunn, Germany)) and the Qubit™ DNA BR assay kit for Qubit 3.0^®^ Fluorometer (Cat# Q32850; Thermo Fisher Scientific). The indexed libraries were pooled for single end sequencing (1 × 75 cycles) on a NextSeq 500 instrument using the v2 150 cycle High Output kit (Cat# 20024906; Illumina) as per manufacturer’s instructions with a coverage of 30 M reads per sample. The base calling and quality scoring were determined using real‐time analysis on board software v2.4.6, while the FASTQ file generation and demultiplexing utilised bcl2fastq conversion software v2.15.0.4.

All reads were aligned to the human genome, build hg38, using align from the Rsubread software package v2.0.1.^33^ Over 94% of reads were successfully mapped for each sample. The number of reads overlapping genes were summarized into counts using featureCounts^34^ from Rsubread. An average of 71% of reads were assigned to genes for each sample. Genes were identified using NCBI RefSeq annotation. Differential expression (DE) analyses were then undertaken using the edgeR^35^ and limma^36^ software packages v3.34.1 and v3.48.3 respectively.

Prior to analysis, all genes with no current symbol, ribosomal RNAs, non–protein coding immunoglobulin genes and haemoglobin genes were removed. Gender‐specific genes including XIST and those unique to the Y chromosome were also removed to avoid gender biases. Expression‐based filtering for lowly expressed genes was then performed using edgeR’s filterByExpr function with default parameters. Library sizes were then normalized using the trimmed mean of the M‐values (TMM) method.^37^

Following filtering and normalization, the data were transformed to log_2_‐counts per million (CPM) with associated precision weights using voom^38^ and the correlation between samples from the same patient estimated using limma’s duplicateCorrelation^39^ function. Sample weights were also calculated using limma’s voomWithQualityWeights^40^ function. Differential expression was then assessed using linear models and robust empirical Bayes moderated *t*‐statistics. To increase precision, the linear models included not only the patient correlation estimate and sample weights, but also incorporated a batch effect correction for cancer type. The false discovery rate (FDR) was controlled below 5% using the Benjamini and Hochberg method.^41^

Kyoto Encyclopaedia of Genes and Genomes (KEGG) pathway analysis, Hallmark gene set analysis and Gene Ontology (GO) were performed to identify signalling pathways associated with the genes differentially expressed between episodes with and without infection. The types of pathway analyses were chosen as indicated for each respective comparison based on how well they represented overall signatures. Analyses of the GO^42^, ^43^ terms and KEGG^44^ pathways were performed using limma’s goana and kegga functions respectively. The analysis of the Hallmark gene sets from the Molecular Signatures Database^45^ was achieved using limma’s fry function.

The mean–difference (MD) plots were generated using limma’s plotMD function and the heatmaps using the pheatmap CRAN software package v1.0.12. The removeBatchEffect function in limma was used to adjust for the effect of cancer type in multi‐dimensional scaling (MDS) plots and heatmaps. Deconvolution of bulk‐RNAseq data to identify immune cell subsets was performed using dtangle.^46^

### Statistical analyses

Ordinary one‐way ANOVA was used to compare immune cell subsets derived from dtangle analysis. Statistical significance was considered when *P* < 0.05.

## Conflict of interest

The authors declare no conflict of interest.

## Author contributions

**Gabrielle M Haeusler:** Conceptualization; Data curation; Funding acquisition; Investigation; Writing – original draft; Writing – review & editing. **Alexandra L Garnham:** Data curation; Formal analysis; Visualization; Writing – original draft. **Connie SN Li‐Wai‐Suen:** Data curation; Formal analysis; Visualization; Writing – original draft. **Julia E Clark:** Project administration; Resources. **Franz E Babl:** Project administration; Resources. **Zoe Allaway:** Project administration; Resources. **Monica A Slavin:** Conceptualization; Funding acquisition; Investigation; Project administration; Writing – original draft. **Francoise Mechinaud:** Conceptualization; Investigation; Resources. **Gordon K Smyth:** Formal analysis; Methodology; Resources; Software; Supervision. **Bob Phillips:** Conceptualization; Investigation; Resources; Writing – original draft. **Karin A Thursky:** Conceptualization; Funding acquisition; Investigation; Project administration; Supervision; Writing – original draft. **Marc Pellegrini:** Conceptualization; Funding acquisition; Investigation; Supervision; Writing – original draft; Writing – review & editing. **Marcel Doerflinger:** Conceptualization; Data curation; Formal analysis; Funding acquisition; Investigation; Writing – original draft; Writing – review & editing.

## Supporting information

Supplementary figures 1–3
Supplementary tables 1–4

## Data Availability

Generated RNA seq data will be deposited in public repository.
