# Supplementary material for: Blood transcriptomics identifies immune signatures indicative of infectious complications in childhood cancer patients with febrile neutropenia
Source: Clin Transl Immunology. 2022 May 17;11(5):e1383. doi: 10.1002/cti2.1383 (PMC9113042; doi:10.1002/cti2.1383)
Supplement: Supplementary file 1 — Supplementary figures 1–3 Supplementary tables 1–4 [file CTI2-11-e1383-s001.pdf]

Blood Transcriptomics identifies immune signatures indicative of infectious complications in childhood cancer patients with febrile neutropenia

Gabrielle M Haeusler, *et al*.

Supporting Information

Supplementary table 1: Definitions of outcomes included in analysis.

Outcome	Definition
**Bacteraemia**	A recognised bacterial pathogen (including organisms associated with mucosal barrier injury in the setting of mucositis or neutropenia) from ≥ 1 blood culture set or common commensals from ≥ 2 blood culture sets drawn on separate occasions.
**A microbiologically documented infection (MDI)**	An infection that was clinically detectable and microbiologically proven.
**Clinically documented infection (CDI)**	A site of infection that is diagnosed but its microbiological pathogenesis either cannot be proven or is inaccessible to examination.
**Unexplained fever**	Any febrile episode without a clinically detectable and microbiologically proven infection.

Supplementary table 2: Immune cell populations (x10^9 L^{-1})

	bacteraemia (n = 9)		MDI (non-bacteraemia) (n = 19)		CDI (n = 12)		unexplained fever (n = 33)		
	**mean**	**SEM**	**mean**	**SEM**	**mean**	**SEM**	**mean**	**SEM**	***P*-value**
**WCC**	0.74444	0.37048	0.88421	0.33901	0.86667	0.20829	0.83939	0.13063	0.99
**ANC**	0.12333	0.08880	0.15211	0.06152	0.11417	0.05770	0.17879	0.04165	0.85
**B cells**	0.00721	0.00282	0.00423	0.00131	0.02053	0.00688	0.01727	0.00476	0.09
**CD8+ T cells**	0.10756	0.06938	0.14613	0.05394	0.14508	0.02971	0.12324	0.02416	0.93
**CD4+ T cells naïve**	0.01665	0.01146	0.11323	0.05417	0.04886	0.02629	0.04144	0.01298	0.22
**CD4+ T cells memory**	0.35521	0.23726	0.24821	0.10318	0.30430	0.07894	0.23189	0.03268	0.81
**CD4+ T cells regulatory**	0.00212	0.00117	0.02222	0.01486	0.00592	0.00401	0.00915	0.00517	0.52
**NK cells**	0.02117	0.00880	0.06388	0.03784	0.04281	0.01759	0.05029	0.01417	0.78
**Monocytes**	0.05043	0.03141	0.04621	0.02114	0.09344	0.03981	0.07745	0.01759	0.57
**Macrophages**	0.01706	0.00855	0.00743	0.00281	0.01360	0.00785	0.01404	0.00382	0.64
**DCs**	0.00002	0.00002	0.00007	0.00006	0.00039	0.00032	0.00055	0.00029	0.47
**Mast cells**	0.00352	0.00198	0.01262	0.00997	0.00764	0.00321	0.00781	0.00241	0.82

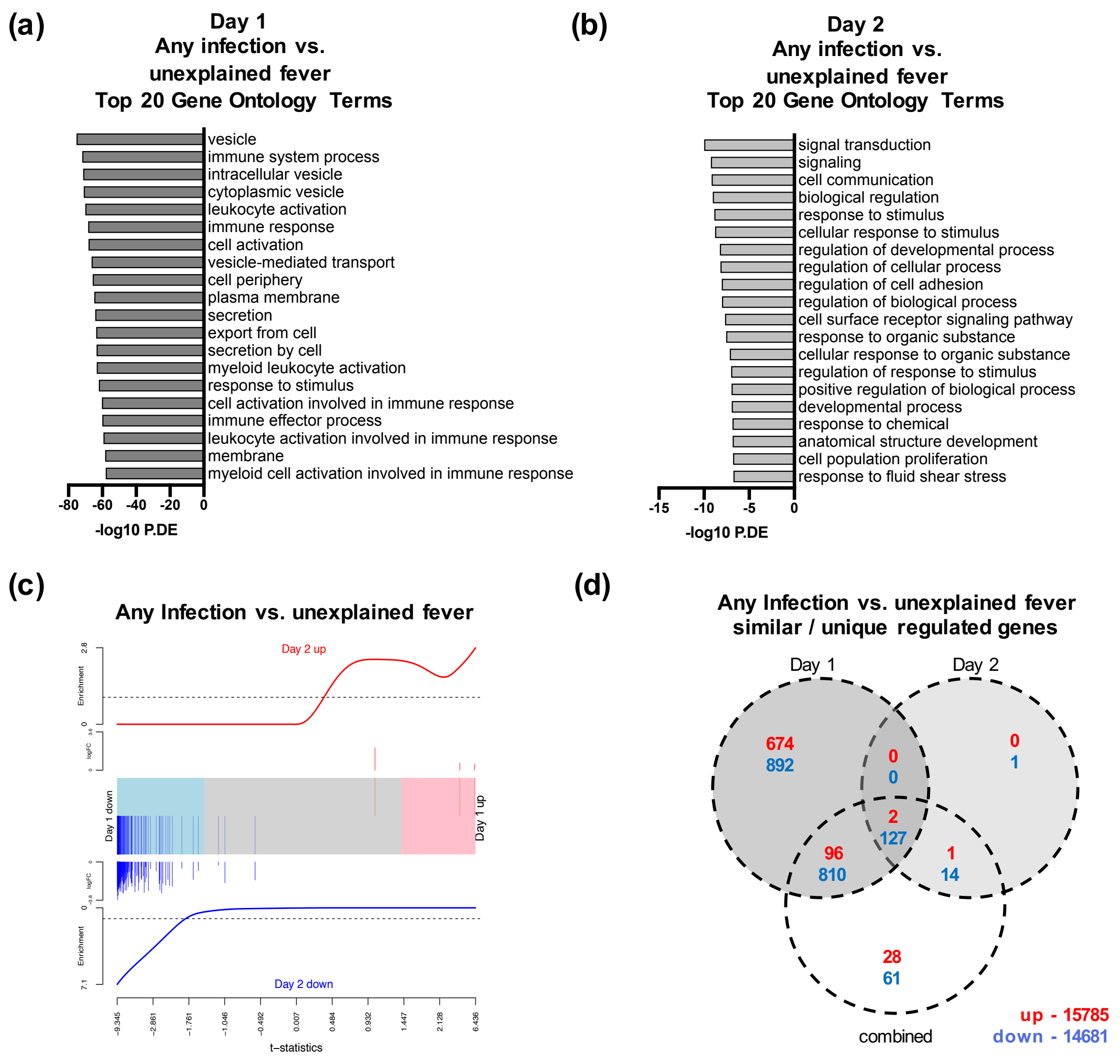

Supplementary figure 1: Gene Ontology and differential gene expression analysis in FN episodes with any infection vs unexplained fever across Day 1 plus Day 2.

(a) Top 20 Gene Ontology pathways over-represented when comparing DE genes in PBMCs from FN episodes with 'any infection' (bacteraemia, MDI and CDI combined) versus unexplained fever at time of hospital admission (Day 1). **(b)** Top 20 Gene Ontology pathways over-represented when comparing DE genes in PBMCs from FN episodes with 'any infection' (bacteraemia, MDI and CDI combined) versus unexplained fever on Day 2. **(c)** Barcode plot analyses of DE genes identified between any infection and unexplained fever identified on Day 1 and Day 2 (gene enrichment test using ROAST method). **(d)** Common and unique DE genes in infection vs. unexplained fever between Day 1, Day 2 or both days combined. Unique DE genes are indicated in the respective circles, while all common DE genes are indicated at the bottom right of the diagram (red, up-regulated; blue, down-regulated).

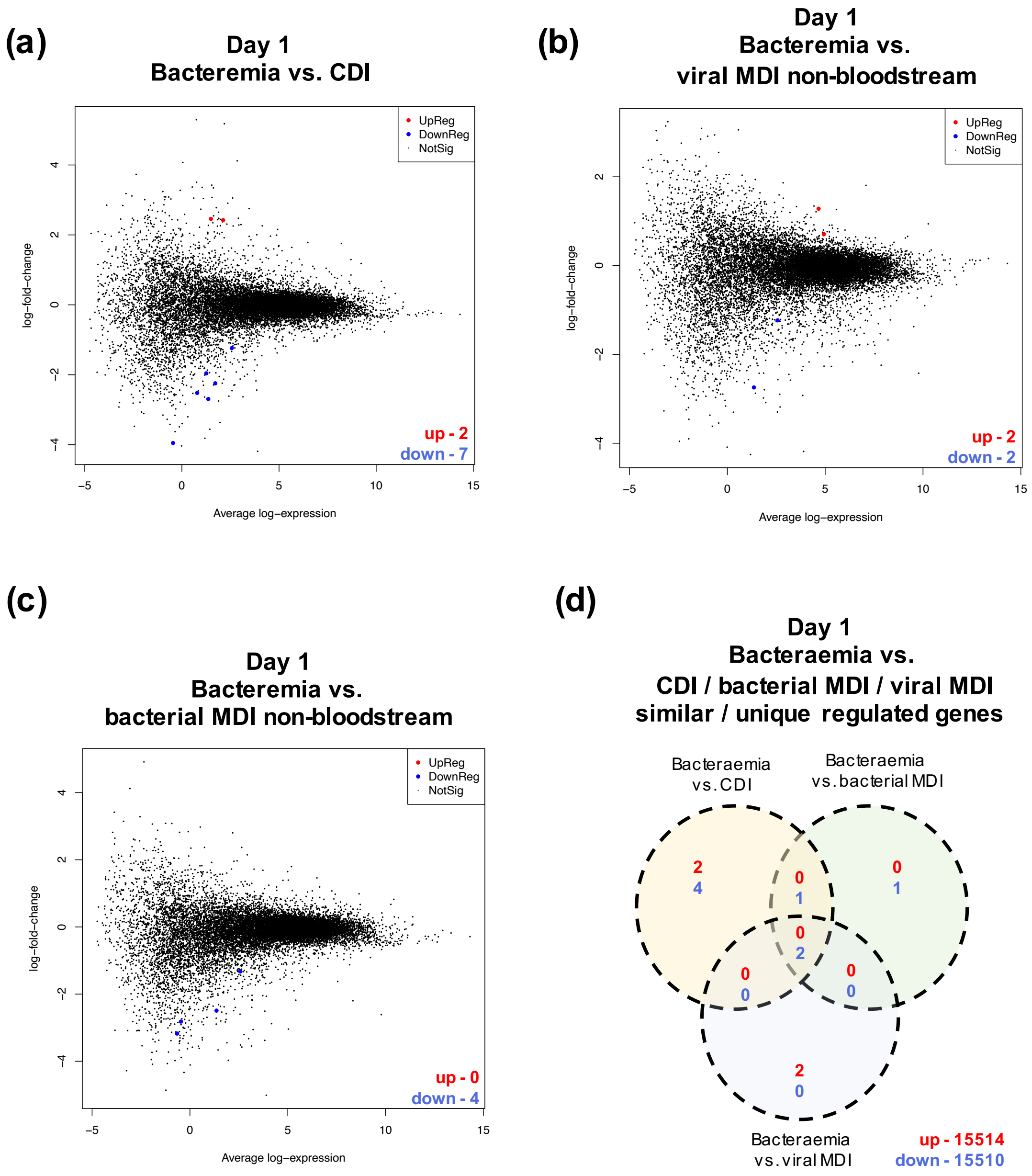

Supplementary figure 2: Differential gene expression for bacteraemia vs. CDI / non-bloodstream bacterial / viral MDI FN episodes at Day 1.

(a) MD plot showing the 7 down-regulated (blue) and 2 up-regulated (red) DE genes in PBMCs from FN episodes with bacteraemia versus CDI causes at time of hospital admission (Day 1). **(b)** MD plot showing the 4 down-regulated (blue) DE genes in PBMCs from FN episodes with bacteraemia versus viral MDI causes at time of hospital admission (Day 1). **(c)** MD plot showing the 4 down-regulated (blue) DE genes in PBMCs from FN episodes with bacteraemia versus bacterial MDI causes at time of hospital admission (Day 1). **(d)** Common and unique DE genes in bacteraemia vs. CDI / non-bloodstream bacterial / viral MDI FN episodes at the time of admission (Day 1). Unique DE genes are indicated in the respective circles, while all common DE genes are indicated at the bottom right of the diagram (red, up-regulated; blue, down-regulated).

Supplementary table 3: Differentially expressed genes in FN episodes with and without bacteraemia at Day 1

Gene	Description	logFC	FDR	B
SNX24	sorting nexin 24	-2.806	0.00004	10.126
RDH10	retinol dehydrogenase 10	-1.288	0.002	6.659
ULK2	unc-51 like autophagy activating kinase 2	-0.862	0.010	4.339
MAP6D1	MAP6 domain containing 1	-2.335	0.010	3.671
ZNF503	zinc finger protein 503	-3.932	0.010	3.454
FXYD1	FXYD domain containing ion transport regulator 1	-2.632	0.010	3.180
KCNC3	potassium voltage-gated channel subfamily C member 3	-1.796	0.010	3.611
IER5L	immediate early response 5 like	-2.496	0.010	3.405
LRRC3	leucine rich repeat containing 3	-2.614	0.010	3.084
FAM87B	family with sequence similarity 87 member B	-2.317	0.010	2.915
LOC100506258	uncharacterized LOC100506258	-1.951	0.011	3.413
NEURL1	Neutralized E3 ubiquitin protein ligase 1	-1.705	0.014	3.149
FAM160A1	family with sequence similarity 160 member A1	-2.801	0.021	2.328
F8	coagulation factor VIII	-1.844	0.024	2.443
SLC17A9	solute carrier family 17 member 9	0.918	0.029	2.039
CABP4	calcium binding protein 4	-1.627	0.040	1.930
LINC00886	long intergenic non-protein coding RNA 886	-3.105	0.040	1.202
GUCY2D	guanylate cyclase 2D, retinal	-3.948	0.042	1.349
FAM221A	family with sequence similarity 221 member A	-1.286	0.042	1.768
FOXN3	forkhead box N3	-0.520	0.043	1.291
PROCA1	protein interacting with cyclin A1	0.840	0.0499	1.384
SYNJ2	synaptojanin 2	0.829	0.0499	1.154
TMX4	thioredoxin related transmembrane protein 4	-0.678	0.0499	1.093
B4GALNT4	beta-1,4-N-acetyl-galactosaminyltransferase 4	-3.011	0.0499	1.027

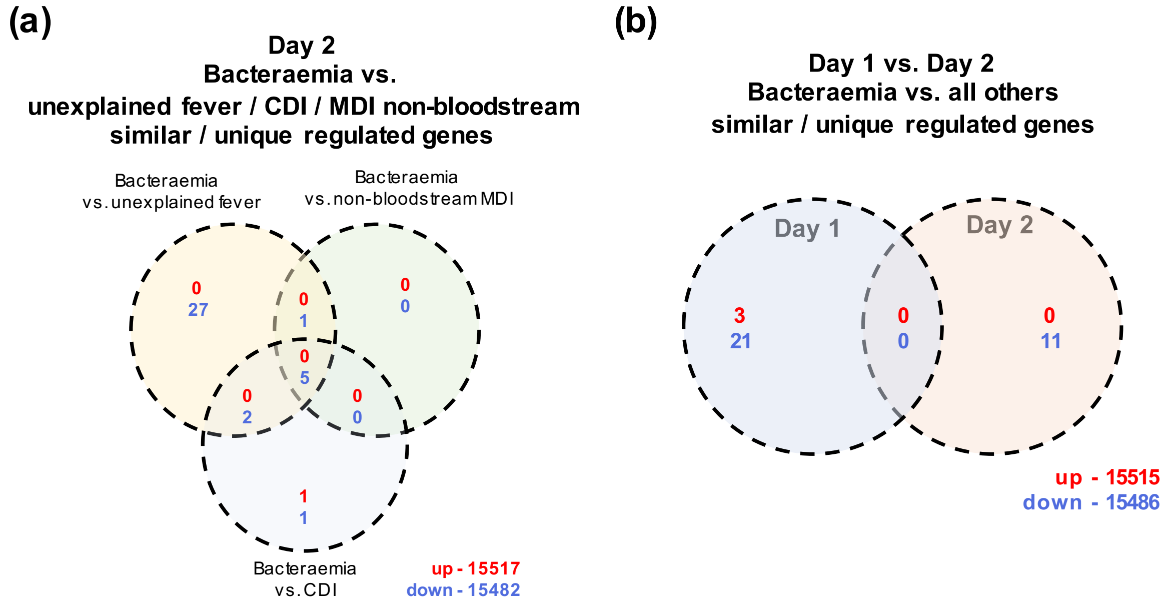

Supplementary figure 3: Bacteraemia versus unexplained ever / CDI / MDI on Day 2 and comparison between Bacteraemia Day 1 and Day 2.

(a) Common and unique DE genes in bacteraemia vs. unexplained fever / CDI / non-bloodstream MDI on Day 2. **(b)** Common and unique DE genes in PBMCs from FN episodes in bacteraemia episodes at Day 1 and Day 2.

Unique DE genes are indicated in the respective circles, while all common DE genes are indicated at the bottom right of the diagram (red, up-regulated; blue, down-regulated).

Supplementary table 4. Day 2 differentially expressed genes identified in episodes with and without bacteraemia

Gene	Description	logFC	FDR	B
BOK	BCL2 family apoptosis regulator BOK	-4.908	0.002	6.449
LOC729867	uncharacterized LOC729867	-5.690	0.002	5.702
SPON2	spondin 2	-5.115	0.007	4.733
NMUR1	neuromedin U receptor 1	-4.698	0.007	4.609
DTHD1	death domain containing 1	-5.341	0.007	4.596
MYRF	myelin regulatory factor	-2.464	0.007	4.229
PDGFRB	platelet derived growth factor receptor beta	-4.545	0.007	4.201
LOC644634	uncharacterized LOC644634	-2.506	0.007	4.154
ST8SIA6	ST8 alpha-N-acetyl-neuraminide alpha-2,8-sialyltransferase 6	-3.598	0.008	4.041
S1PR5	sphingosine-1-phosphate receptor 5	-4.673	0.024	2.803
MMP23B	matrix metallopeptidase 23B	-4.471	0.039	2.035
